# Cardiovascular safety of fixed-dose extended-release naltrexone/bupropion in clinical practice

**DOI:** 10.1016/j.obpill.2025.100169

**Published:** 2025-02-17

**Authors:** Michael Kyle, Dustin Burns, Catherine Rogers Murray, Heather Watson, Jeff Swaney, Samuel Spevack, Megan Leonhard, Michael Simon, Emma Moynihan, Kate L. Lapane, Shirley V. Wang, Craig L. Longo, Mary E. Ritchey, David D. Dore

**Affiliations:** a Currax Pharmaceuticals, LLC, Brentwood, TN, USA; b Exponent, Inc., Irvine, CA, USA; c Exponent, Inc., Natick, MA, USA; d Exponent, Inc., Bellevue, WA, USA; e Exponent, Inc., Bowie, MD, USA; f Consultant to Exponent, Inc., Worcester, MA, USA; g Division of Pharmacoepidemiology and Pharmacoeconomics, Brigham and Women's Hospital, Boston, MA, USA; h Department of Medicine, Harvard Medical School, Boston, MA, USA; i Emergency Department, St. Luke's Hospital, New Bedford, MA, USA; j CERobs Consulting, LLC, Philadelphia, PA, USA; k Department of Health Services, Policy & Practice, Brown University School of Public Health, Providence, RI, USA

**Keywords:** Antiobesity medication, Cardiovascular safety, Fixed-dose extended-release combination of naltrexone/bupropion, Obesity

## Abstract

**Background:**

The fixed-dose extended-release combination of naltrexone/bupropion (NB-ER) is indicated to treat overweight and obesity in adults as an adjunct to a reduced-calorie diet and increased physical activity. This study compared the rate of major adverse cardiovascular events (MACE) and its components (nonfatal acute myocardial infarction [AMI], nonfatal stroke, and cardiovascular death) between patients initiating NB-ER and those initiating lorcaserin (removed from US market in 2020; included as active comparator to minimize possible confounding by indication) in routine clinical practice.

**Methods:**

This was a retrospective cohort study with a new-user, active-comparator design. Patients initiating NB-ER or lorcaserin were identified using Arcadia Data Research electronic health records, including insurance claims (June 2012–February 2020). Incidence rate ratios were estimated, and adjusted hazard ratios (aHRs) with 95 % confidence intervals (CIs) were estimated using a propensity score (PS)-weighted Cox proportional hazard model in an intention-to-treat analysis.

**Results:**

Patients initiating NB-ER (n = 12 475) or lorcaserin (n = 12 171) were followed for a mean observation period of 4.7 years. After PS weighting, baseline comorbidities, concomitant medications, lifestyle factors, and clinical measures were balanced between cohorts. MACE incidence was 0.77/1000 person-years for NB-ER and 1.03/1000 person-years for lorcaserin. Compared to lorcaserin, patients initiating NB-ER had statistically similar rates of MACE (aHR, 0.76; 95 % CI, 0.48–1.22), nonfatal AMI (aHR, 0.74; 95 % CI, 0.45–1.23), and nonfatal stroke (aHR, 1.05; 95 % CI, 0.34–3.22). No deaths were observed within 30 days of an AMI or stroke.

**Conclusion:**

Patients initiating NB-ER compared with lorcaserin were not at an increased risk of MACE or its components. Conclusions from this study must be interpreted in the context of certain assumptions related to PS methodology and use of lorcaserin as an active comparator. Causal interpretations for the cardiovascular safety of NB-ER should be evaluated further in a prospective, randomized, blinded, controlled clinical trial.

## Highlights

• EHR data showed fixed-dose extended-release naltrexone/bupropion (NB-ER) vs lorcaserin did not have increased CV events.
• Patients initiating NB-ER vs lorcaserin did not experience increased rates of nonfatal stroke or nonfatal AMI.
• No deaths were observed within 30 days of an AMI or stroke event among patients initiating NB-ER.

## Graphical abstract

**Figure:**
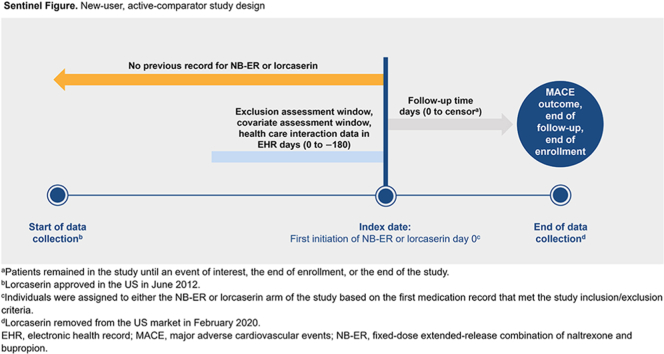

## 1 Introduction

Obesity is a chronic disease that affects approximately 76 million people in the US [1]. Treatment options include changes to diet and exercise as well as adjunctive medical interventions, such as surgical procedures and medications [2,3]. The fixed-dose extended-release combination of naltrexone and bupropion (NB-ER) was approved by the US Food and Drug Administration (FDA) in 2014 and by the European Medicines Agency in 2015 for the treatment of obesity in adults with a body mass index (BMI) ≥30 kg/m^2^ or with a BMI ≥27 kg/m^2^ to <30 kg/m^2^ and ≥1 weight-related comorbidity (eg, type 2 diabetes mellitus, dyslipidemia, hypertension) [4,5]. NB-ER is indicated for use as an adjunct to a reduced-calorie diet and increased physical activity [4,5].

Lorcaserin, a selective serotonin 5-HT_2C_ agonist, was approved in the US for the same indication as NB-ER [6]. In a lorcaserin cardiovascular (CV) safety and efficacy trial, there was no difference in CV risk for lorcaserin relative to placebo [7]. Lorcaserin was withdrawn from the market in 2020 due to cancer safety concerns [8].

Some antiobesity medications (AOMs) have CV adverse effects, including increased blood pressure, increased heart rate, or occurrence of major adverse CV events (MACE; composite of nonfatal acute myocardial infarction [AMI], nonfatal stroke, and CV death) [9]. Regulatory authorities withdrew several AOMs from the market due to adverse effects, including CV toxicity [10]. AOMs must often be continued long-term [2,11], and characterizing the long-term CV safety of AOMs is critical. The LIGHT trial was a phase 3b randomized controlled trial (RCT; N = 8910) designed to assess the occurrence of MACE in patients with overweight or obesity at an increased risk of adverse CV outcomes treated with NB-ER [12]. Although this trial was terminated early, this termination was not related to any safety concerns [12]. The 25 % and 50 % interim analyses showed no evidence of increased CV risk in patients treated with NB-ER vs placebo [12,13]. However, because of the unanticipated early termination of the trial, noninferiority could not be confirmed, and the CV safety of NB-ER requires further study, especially in the setting of routine clinical practice.

We conducted a cohort study using electronic medical records and health insurance claims (electronic health records [EHRs]) with the primary objective of comparing the incidence of MACE between patients initiating NB-ER vs lorcaserin, another oral AOM [6,8]. We also report results from an analysis in which we emulated a published randomized trial of lorcaserin vs placebo on the occurrence of MACE to evaluate the suitability of the EHR data and primary methods.

## 2 Methods

### 2.1 Study design

We used a retrospective, new-user, active-comparator cohort design within linked EHR and mortality data. New-user, active-comparator cohort designs reduce confounding and avoidable design flaws such as immortal time bias [6]. Both NB-ER and the active comparator, lorcaserin, were dispensed to individuals for the same indication [14], reducing concerns about important differences in risk factors before covariate adjustment. Lorcaserin was previously used as an active comparator as a clinically relevant alternative to NB-ER but is no longer on the market due to safety concerns [15].

### 2.2 Data source

The study's data source was Arcadia Data Research (Arcadia Solutions, Boston, MA, USA), a compilation of EHRs, including linked claims for approximately 50 % of individuals, from multiple health care systems, academic medical centers, ambulatory and primary care facilities, hospitals, and other providers. At the time of data extraction, Arcadia's data included 135 million people in the US, of whom 75 million had available deidentified information for research. For the primary objective, Arcadia data were linked to Datavant's Death Index. Datavant, a private data curator based in the US, specializes in linking deidentified health data in a manner compliant with the Health Insurance Portability and Accountability Act (HIPAA). The death information comes from the Social Security Administration's Death Master File, private obituaries, newspapers, and claims data [[16], [17], [18]].

Medications were mapped to the Hierarchical Ingredient Code Lists from First Databank [19]; laboratory results to Logical Observation Identifiers Names and Codes, Systematized Nomenclature of Medicine-Clinical Terms; procedures to Current Procedural Terminology and Healthcare Common Procedure Coding System codes; and conditions to International Classification of Diseases (ICD)-9/10 codes.

Confidentiality and deidentification of patient records were always maintained. All analyses were performed in accordance with applicable laws and regulations.

### 2.3 Source population

The source population was composed of adults ≥18 years of age who received NB-ER or lorcaserin and interacted with a health care system that contributed to the Arcadia data. The study medication was restricted to NB-ER because concomitant use of naltrexone and bupropion is not indicated for the treatment of obesity. Lorcaserin was included in this study since, in the context of new-user designs, active comparators increase comparability between patient groups, thus helping to minimize possible confounding by indication [15]. Confounding by indication occurs when the condition that led to the choice of treatment is associated with the risk of the outcome, making it challenging to discern whether the treatment or the underlying condition is responsible for any potential effect. Additionally, lorcaserin had a similar indication and no effect on the occurrence of MACE compared to placebo in a noninferiority analysis of a large, randomized CV outcomes trial [6,8]. Initiation dates were between June 1, 2012 (FDA approval of lorcaserin for treatment of obesity [6]), and February 12, 2020 (date lorcaserin was removed from the US market due to potential carcinogenicity [20]).

The index date (date of study entry) was defined as the first date of a medication record for NB-ER or lorcaserin preceded by ≥ 1 record from 180 to 360 days prior to the index date and ≥1 additional record within the 180 days prior to the index date (baseline period). Requiring pre-initiation health care interactions limited the study population to individuals with longitudinal data and with the expectation of sufficient clinical information to define study variables. We excluded patients without a BMI measurement in the baseline period and those with a history of epilepsy, bulimia, anorexia, a weight-loss procedure, or opioid use (Supplemental Table 1). The medicine initiated on the first qualifying index date defined exposure (NB-ER or lorcaserin), and patients were excluded if they received both NB-ER and lorcaserin on the index date (N = 113).

### 2.4 Covariates

A wide range of demographic, health history, and medical characteristics were defined in the baseline period for each individual. These prespecified variables are listed in Supplemental Table 2. Additionally, we identified the 100 most prevalent conditions, procedures, and medications from the EHR data. These variables were used in estimating propensity scores (PSs) to decrease the risk of unmeasured confounding by including a large set of covariates that may serve as proxies for unmeasured variables.

### 2.5 Outcomes and follow-up time

**Fig. 1:**
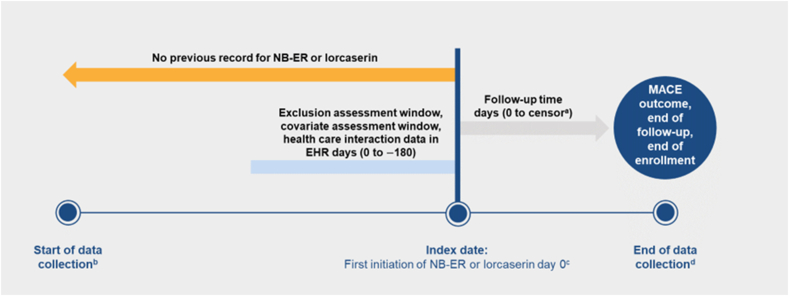
New-user, active-comparator study design ^a^Patients remained in the study until an event of interest, the end of enrollment, or the end of the study. ^b^Lorcaserin approved in the US in June 2012. ^c^Individuals were assigned to either the NB-ER or lorcaserin arm of the study based on the first medication record that met the study inclusion/exclusion criteria. ^d^Lorcaserin removed from the US market in February 2020. EHR, electronic health record; MACE, major adverse cardiovascular events; NB-ER, fixed-dose extended-release combination of naltrexone and bupropion.

The primary study endpoint was MACE, defined as the composite of medically attended nonfatal AMI, medically attended nonfatal stroke, or CV death (Fig. 1). Within Arcadia EHR data, each occurrence of an ICD-9/10 code for AMI or stroke was considered potentially indicative of an event, and the date corresponding to the code in the EHR was denoted as the tentative event date. Each potential case of MACE and its event date were then adjudicated by a US-licensed and practicing emergency physician (CLL) masked to NB-ER or lorcaserin initiation. The physician consultant reviewed the chronological listings of each potential case's individual EHR to determine whether there was clinical evidence consistent with the event having occurred. If the initial EHR-based definition did not satisfy the physician consultant's clinical assessment of the case status and its date, the event was reclassified as a non-case for analysis and/or the date of occurrence was adjusted. Adjudicated events were excluded when (1) the events were determined not to be acute (58 % of AMI events, 74 % of stroke events); (2) the physician consultant was unable to confirm the potential AMI or stroke given the available data; or (3) the event occurred after a censoring event.

CV death was defined as confirmed cases from the EHR that had a Datavant death record within 30 days of the AMI or stroke, inclusive of the date of the AMI or stroke. To mitigate linkage inaccuracies or misattributions of a death between Datavant's Death Index and Arcadia EHR data, each AMI, stroke, and CV death underwent adjudication by the physician consultant or the senior author. Only cases with death records that were consistent with the EHR data (ie, data stopped accumulating after the date of death) were classified as fatal CV events.

Follow-up time was calculated separately for each study endpoint using an intention-to-treat (ITT) approach. Patients were followed starting the day after initial exposure until the earliest of (1) the study endpoint; (2) the end of activity in the EHR plus 6 months; or (3) the end of the study period (ie, December 2022). Because EHR data do not contain information on patients’ active/inactive status within the health care system, we defined end of enrollment as the date of the last observed interaction in the health care system plus an additional 6 months as an approximation of time at-risk, assuming not all individuals change health systems (and, therefore, potentially leave the health care systems that contributed to Arcadia data) on the date of their last interaction with their current health system.

### 2.6 Statistical analysis

#### 2.6.1 Multiple imputation and primary analyses

Because some missing values of data in EHRs are a persistent problem, multiple imputation by chained equations was used to impute missing covariate values [21]. This procedure involves fitting a series of multivariable regression models to predict values of missing variables given the observed data. When data are missing at random, multiple imputation preserves sample size and mitigates bias that can arise when only patients with complete data are included [21,22]. Variables included in the imputation model represented demographic information, comorbid conditions, concomitant medications, laboratory results, and vital signs. An interaction between the year of the index date (medication initiation) and the first 3 zip code digits was included to account for geographic or health-system effects on missingness. Ten imputed datasets were generated with randomly selected, independently drawn values predicted by the imputation models. Then, analyses to estimate the effect of NB-ER vs lorcaserin on MACE outcomes were run within each imputed dataset, and the results were combined as the average of within-dataset values to generate an estimate. Corresponding 95 % CIs were computed using Rubin's rules [22,23]. The crude incidence rates of outcomes were estimated for each primary and secondary endpoint and separately for patients initiating NB-ER and lorcaserin.

#### 2.6.2 Propensity score

This study used PS weighting to balance covariates between the exposure groups and allow for causal interpretation within the context of appropriate design and data. The PS is the predicted probability of receiving treatment relative to the comparator, conditional on inclusion in the study and baseline covariates [24]. PSs were estimated via logistic regression modeling from the prespecified covariates and the 100 most prevalent conditions, procedures, and medications. We used a PS weighting method that is analogous to pair-matching and that results in better statistical efficiency and balance between the treatment cohorts [25]. This technique estimates the average treatment effect among individuals whose PS was present in both the NB-ER and lorcaserin groups, providing a proxy for clinical eligibility for either drug (ie, empirical equipoise) [26]. The PS weights were trimmed at the 1 % tails, removing patients who were least likely to be comparable between the cohorts on unmeasured confounders and reducing undue influence of extreme values [25]. Standardized differences in means and proportions were used to assess the covariate balance between treatment cohorts after applying the trimmed PS weights. For each study endpoint, we estimated adjusted (weighted) hazard ratios (aHRs) and 95 % CIs using Cox proportional hazards modeling.

### 2.7 Additional analysis

An RCT emulation analysis was conducted to assess the utility of the EHR data by benchmarking the results of this analysis against results from the CAMELLIA-TIMI 61 trial [7]. The CAMELLIA-TIMI 61 trial compared the risk of MACE for lorcaserin vs placebo [7]. We compared lorcaserin to patients receiving weight-loss counseling (a proxy for placebo) after applying the trial eligibility criteria to the EHR cohort. The weight-loss counseling group comprised individuals who did not start lorcaserin therapy but who had evidence of weight-management education classes, nutrition counseling, or behavior counseling for obesity to mimic the patient engagement with weight-management activities present in the trial. The emulation and concordance metrics for agreement between the EHR analysis and RCT results were adapted from the RCT DUPLICATE initiative [27]. For the emulation analysis, we used comparable methods for characterizing exposure and outcomes, for estimating the PSs, and for the principal analysis relative to the primary analysis.

## 3 Results

**Fig. 2:**
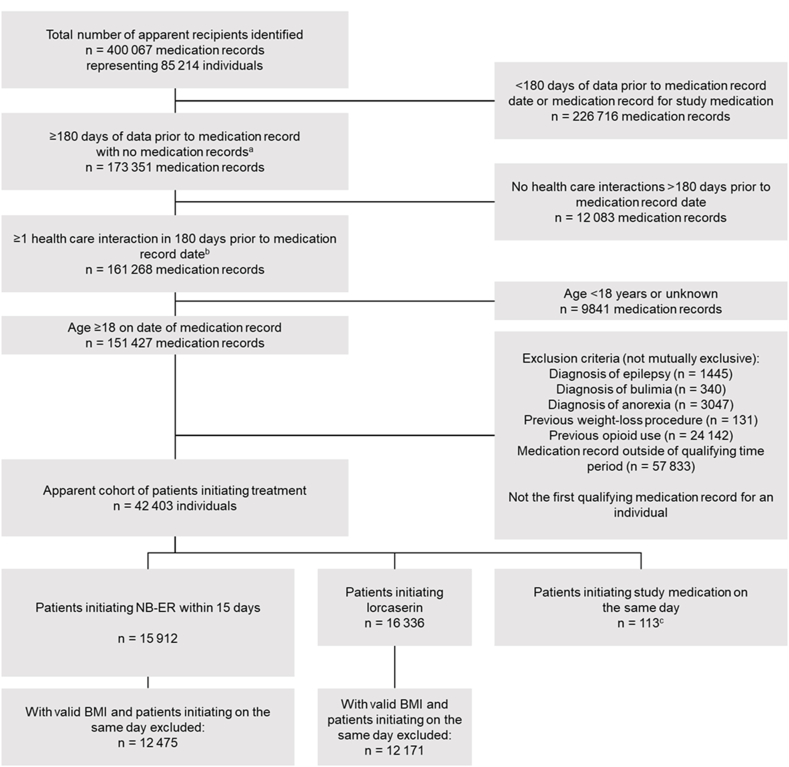
Primary study cohort ^a^Defined as ≥1 record of any type ≤180 days prior to the date of qualifying medication record. ^b^Defined as ≥1 record of health care interactions ≤180 days prior to the date of medication record. ^c^These individuals were excluded from the analysis. BMI, body mass index; NB-ER, fixed-dose extended-release combination of naltrexone and bupropion.

There were 12 475 eligible patients initiating NB-ER and 12 171 patients initiating lorcaserin (Fig. 2). Before PS weighting, the NB-ER and lorcaserin cohorts had similar demographics. For both cohorts, the mean age was approximately 48 years, 82 % were female, and two-thirds of the patients were White (Table 1). The prevalence of comorbid conditions was also similar between the 2 cohorts; the largest differences were observed for major depressive disorder (NB-ER, 11.4 %; lorcaserin, 7.2 %) and hypertension (NB-ER, 34.3 %; lorcaserin, 38.5 %). After PS weighting, the distributions of baseline covariates were nearly identical for NB-ER and lorcaserin.

**Table 1:** Patients initiating NB-ER and lorcaserin: baseline demographics and clinical characteristics.

	Unweighted^a^		Weighted^b^		
	**NB-ER (n = 12 475)**	**Lorcaserin (n = 12 171)**	**NB-ER (n = 12 364)**	**Lorcaserin (n = 12 035)**	**Standardized difference**
**Age, years, mean (SD)**	47.6 (11.9)	48.1 (12.2)	47.9 (10.1)	47.9 (10.4)	0.002
**Sex**					
Female	82.3	81.8	81.7	81.7	−0.002
**Race**					
White	67.1	62.3	65.0	65.0	−0.001
Black or African American	9.2	14.7	11.2	11.3	−0.002
Asian	0.8	1.2	0.9	0.9	−0.002
Other/unknown^c^	22.9	21.8	22.9	22.8	0.003
**Ethnicity**					
Not Hispanic/Latino	68.3	69.2	68.2	68.1	0.003
Hispanic/Latino	6.3	7.2	6.8	6.9	−0.005
Unknown^c^	25.3	23.5	25.0	25.0	0.000
**Diagnoses from EHR**^d^					
Hypertension	34.3	38.5	36.2	36.1	0.001
Type 2 diabetes mellitus	11.6	14.0	12.6	12.6	−0.002
Heart failure	0.6	0.7	0.6	0.6	−0.009
Unstable angina	3.7	3.8	3.7	3.7	0.001
CKD	1.6	2.0	1.8	1.9	−0.005
OSA	7.7	7.8	7.8	7.8	−0.001
COPD	1.3	1.8	1.5	1.5	0.000
Bipolar disorder	1.6	1.9	1.7	1.7	−0.005
MDD	11.4	7.2	8.4	8.4	0.001
**Laboratory results and vitals, mean**^e^					
BMI, kg/m^2^	37.3	37.4	37.3	37.3	−0.003
Glucose, mg/dL	103.7	105.1	104.5	104.3	0.004
Total cholesterol, mg/dL	190.4	189.3	190.0	189.9	0.002
LDL, mg/dL	111.0	109.6	110.5	110.4	0.002
HDL, mg/dL	57.6	57.0	57.3	57.3	0.003
Triglycerides, mg/dL	139.3	138.1	139.1	139.2	−0.001
Systolic BP, mm Hg	125.4	125.5	125.4	125.4	0.002
Diastolic BP, mm Hg	79.1	78.9	79.0	78.9	0.003
HbA1c, %	5.8	5.8	5.8	5.8	0.003
GFR, mL/min/1.73 m^2^	59.7	59.6	59.7	59.7	0.003
Heart rate, bpm	79.9	79.4	79.6	79.6	0.003
Serum creatinine, mg/dL	0.8	0.8	0.8	0.8	−0.001

All data presented as % unless otherwise noted.

BMI, body mass index; BP, blood pressure; bpm, beats per minute; CKD, chronic kidney disease; COPD, chronic obstructive pulmonary disease; EHR, electronic health record; GFR, glomerular filtration rate; HbA1c, hemoglobin A1c; HDL, high-density lipoprotein; LDL, low-density lipoprotein; MDD, major depressive disorder; MI, multiple imputation; NB-ER, fixed-dose extended-release combination of naltrexone and bupropion; OSA, obstructive sleep apnea; PS, propensity score.

a The unweighted distributions were computed by taking the mean across 10 MI datasets prior to PS weights.

b The values presented are the mean PS-weighted values across 10 MI datasets, each including patients between the first and 99th percentile of the PS. Patients whose PS were in the first percentile and 99th percentile were excluded (trimmed).

c The “unknown” value was present by default in the Arcadia data. As a result, no observations were missing values.

d We assumed the presence of a code implied that an individual had the condition and the absence of a code indicated they did not have the condition.

e Ascertained from the 365 days prior to (and including) the index date.

Patients initiating each treatment had similar mean durations of follow-up from the index date (NB-ER, 4.7 years; lorcaserin, 4.7 years). The maximum follow-up was 8.1 years for NB-ER (25th percentile: 3.6 years; 75th percentile: 5.8 years) and 9.4 years for lorcaserin (25th percentile: 3.3 years; 75th percentile: 6.1 years). The percentage of patients initiating NB-ER with a follow-up of >5 years was 45.5 % and for patients initiating lorcaserin it was 40.1 %.

Over the course of follow-up, patients initiating NB-ER had 31 MACE outcomes vs 40 for patients initiating lorcaserin, 26 AMI outcomes vs 36, and 6 nonfatal stroke outcomes vs 5 (Table 2). There were no deaths observed in the linked data within 30 days of an AMI or stroke event. The crude incidence rate of MACE was 0.77/1000 person-years for patients initiating NB-ER vs 1.03/1000 person-years for patients initiating lorcaserin (rate difference, −0.27/1000 person-years; rate ratio, 0.74). After PS weighting, the aHR of MACE comparing NB-ER vs lorcaserin was 0.76 (95 % CI, 0.48–1.20). The aHR of nonfatal AMI was 0.74 (95 % CI, 0.45–1.23), and the aHR of nonfatal stroke was 1.05 (95 % CI, 0.34–3.22).

**Table 2:** Crude and adjusted incidence of MACE among patients initiating NB-ER vs lorcaserin.

	Number of outcomes, NB-ER	Number of outcomes, lorcaserin	Mean follow-up, days, NB-ER; lorcaserin	Crude IRD per 1000 PY	Crude IRR	HR (95 % CI)^a^
**Primary endpoint**^c^	31	40	1705; 1702	−0.27	0.74	0.76 (0.48–1.22)
**Secondary endpoint**						
AMI	26	36	1705; 1703	−0.28	0.69	0.74 (0.45–1.23)
Stroke	6	5	1707; 1705	0.00	1.00	1.05 (0.34–3.22)
CV death^d^	0	0	1709; 1707	b	b	b

Models were PS-weighted, and estimates in each of the 10 imputed datasets were combined using Rubin's rules.

Individuals with the outcome of interest during the baseline period were excluded from the incidence analysis, including counts of the events.

IRD, IRR, and HR compared the patients initiating NB-ER and lorcaserin.

The PS was calculated, and trimming was undertaken in each of the 10 imputation datasets separately. Therefore, the event counts and follow-up time are computed in the PS-weighted cohort and mean across each of the 10 imputations.

AMI, acute myocardial infarction; CV, cardiovascular; HR, hazard ratio; IRD, incidence rate difference; IRR, incidence rate ratio; ITT, intention-to-treat; MACE, major adverse cardiovascular events; NB-ER, fixed-dose extended-release combination of naltrexone and bupropion; PS, propensity score; PY, person-year.

a Accounts for PS weights.

b The mean number of events across the 10 imputation datasets was <1.

c The primary endpoint was MACE, which consisted of nonfatal AMI, nonfatal stroke, and CV death.

d CV death was defined as death within 30 days and inclusive of an AMI or stroke.

### 3.1 Additional analysis

For the emulation analysis, 999 patients initiating lorcaserin and 163 075 patients initiating weight-loss counseling were identified. After PS weighting, all covariates were balanced. The aHR of modified MACE (combination of AMI and stroke due to a lack of linkage to death information for this analysis) in the emulation study was 0.84 (95 % CI, 0.46–1.54), and the hazard ratio of MACE (nonfatal AMI, nonfatal stroke, and CV death) in the CAMELLIA-TIMI 61 trial was 0.99 (95 % CI, 0.85–1.14). The hazard ratios of AMI were similar between the studies (CAMELLIA-TIMI 61 trial: aHR, 0.99 [95 % CI, 0.82–1.19]; emulation study: aHR, 1.02 [95 % CI, 0.61–1.71]; Table 3). Concordance of results for stroke could not be evaluated, as there were no stroke events among the patients initiating lorcaserin in the EHR cohort.

**Table 3:** Standardized difference agreement for the RWD study and CAMELLIA-TIMI 61 trial.

	HR (95 % CI)			
	CAMELLIA-TIMI 61 trial	RWD emulation	Statistical significance agreement^a^	Estimate agreement^b^
**AMI**	0.99 (0.82–1.19)	1.02 (0.61–1.71)	Yes, with regard to statistical significance	Yes
**Strok**e^c^	0.86 (0.64–1.15)^c^	NA	NA	NA
**MACE**^d^	0.99 (0.85–1.14)	NA	NA	NA
**MACE**^e^	NA	0.84 (0.46–1.54)	NA	NA

Findings for the CAMELLIA-TIMI 61 trial can be found in Bohula et al [7].

NA indicates differences between CAMELLIA-TIMI 61 trial and this RWD emulation comparison.

AMI, acute myocardial infarction; CV, cardiovascular; HR, hazard ratio; MACE, major adverse cardiovascular events; NA, not applicable; RWD, real-world data.

a Statistical significance agreement is defined as a statistically significant RWD effect estimate in the same direction as the trial effect estimate.

b Estimate agreement defined as the RWD HR within the 95 % CI for the trial estimate.

c Estimates for stroke could not be reliably calculated in the RWD study due to the absence of events in the lorcaserin arm.

d MACE was defined in the CAMELLIA-TIMI 61 trial as follows: nonfatal AMI, stroke, or CV death.

e MACE was defined in the RWD study as follows: nonfatal AMI or stroke. Direct comparisons could not be made due to differences in definition.

## 4 Discussion

This study characterized the long-term, real-world CV safety of NB-ER. The overall incidence of MACE, and each component outcome, was low among patients initiating NB-ER and lorcaserin. The rate of MACE among patients initiating NB-ER vs lorcaserin was statistically similar over a mean follow-up of 4.7 years. Similar results were observed for nonfatal AMI, nonfatal stroke, and CV death. The findings of the emulation analysis in the EHR data were consistent with evidence from the CAMELLIA-TIMI 61 trial [7].

The low rate of MACE among patients initiating NB-ER, which was driven by the comparatively more common nonfatal AMI, is consistent with the age (mean < 50 years) and sex distribution (approximately two-thirds female) of the study population. Similar CV incidence estimates have been reported for other AOMs, including orlistat [28], phentermine-topiramate [29], liraglutide [30], and semaglutide [31].

We did not observe an increased rate of AMI among patients initiating NB-ER compared to lorcaserin. That the emulation study demonstrated agreement with the CAMELLIA-TIMI 61 trial results on the available data (nonfatal AMI; stroke data were sparse) [7] provides assurance that the EHR data and methodological approach were appropriate for the primary analysis. As the population included in our analysis is more heterogenous in terms of sociodemographics, age, and comorbid conditions, and our dataset has a longer mean follow-up time (4.7 years) vs the CAMELLIA-TIMI 61 trial (2.3 years), the results of these analyses complement the available trial evidence [32].

### 4.1 Limitations

There are several limitations to this study, including confounding by factors not included in EHRs, such as socioeconomic status (eg, income, education, employment). Electronic medical records and insurance claims data may be subject to biases related to misclassification, measurement, and missing data. Other possible sources of bias may be a health system's medical coding policies, the lack of interoperability between disparate health record systems, health care system staffing, and reimbursement practices. Additionally, this study of electronic medical records and health insurance claims data must be interpreted in the context of certain assumptions related to PS methods, including positivity, consistency, lack of selection bias, no unmeasured confounding, no measurement error, correct specification of PS weights, and correct specification of the outcome model [33,34]. However, this study included rich clinical information from EHRs. We defined a large set of covariates, which may proxy-adjust for confounders that are not directly measured in the EHRs [35,36]. There were also missing data for multiple covariates, which was addressed by applying multiple imputation methods.

We found that codes indicating AMI or stroke are imperfect measures of the acute occurrence of these events on the date of record. We expect that the clinical adjudication of outcomes in the primary analysis resulted in a low probability of including false-positive cases in the analysis. However, other sources of misclassification may remain, including outcome events treated in health systems not represented in the Arcadia data.

Identifying CV death proved challenging, as ambiguities in the privacy-preserving data linking and attribution of death records to unique persons were present, likely resulting in incomplete capture of deaths [17]. Manual adjudication of apparent deaths relative to the Arcadia data and exclusion of one-to-many matches between deaths and individuals prevented incorrect deaths from being included in the analysis. Similarly, our study focused on long-term CV safety, but we did not specifically distinguish events that occurred while on therapy vs after discontinuation.

## 5 Conclusions

In this analysis of EHR data, our findings suggest that patients initiating NB-ER were not at an increased risk of MACE or its components compared to patients initiating lorcaserin. The results of this study should be interpreted in the context of specific assumptions related to PS weighting and the use of lorcaserin as an active comparator. However, the results of the emulation analysis largely aligned with the CAMELLIA-TIMI 61 trial, which supports the validity of the methodology and data sources used. While a particular strength of this study was that the EHR information provided a vast array of variables for inclusion in the PS development, a prospective, randomized, blinded, controlled clinical trial with independent adjudication and applicable statistical analysis may provide the basis for definitive conclusions about the causal interpretations of the study medications on the CV outcomes of interest.

**Key takeaways**

• Patients initiating NB-ER compared with lorcaserin were not at an increased risk of MACE or its components
• The emulation analysis suggests that the data sources and methodological approaches were appropriate
• While a particular strength of this study was that the EHR information provided a vast array of variables for inclusion in the PS development, a prospective, randomized, blinded, controlled clinical trial with independent adjudication and applicable statistical analysis may provide the basis for definitive conclusions about the causal interpretations of the study medications on the CV outcomes of interest

## CRediT authorship contribution statement

Michael Kyle: Methodology, Investigation, Writing – Original Draft, Writing - Review & Editing, Visualization, Supervision, Funding Acquisition. Dustin Burns: Data Curation, Formal Analysis, Methodology, Investigation, Writing – Original Draft, Writing - Review & Editing, Validation, Visualization. Catherine Rogers Murray: Methodology, Investigation, Writing – Original Draft, Writing - Review & Editing, Visualization, Project Administration. Heather Watson: Methodology, Investigation, Writing – Original Draft, Writing - Review & Editing, Visualization. Jeff Swaney: Methodology, Investigation, Writing – Original Draft, Writing - Review & Editing, Visualization. Samuel Spevack: Methodology, Investigation, Writing – Original Draft, Writing - Review & Editing, Visualization. Megan Leonhard: Methodology, Investigation, Writing – Original Draft, Writing - Review & Editing, Visualization. Michael Simon: Methodology, Investigation, Writing – Original Draft, Writing - Review & Editing, Visualization. Emma Moynihan: Methodology, Investigation, Writing – Original Draft, Writing - Review & Editing, Visualization. Kate L. Lapane: Methodology, Investigation, Writing – Original Draft, Writing - Review & Editing, Visualization. Shirley V. Wang: Methodology, Investigation, Writing – Original Draft, Writing - Review & Editing, Visualization. Craig L. Longo: Methodology, Investigation, Writing – Original Draft, Writing - Review & Editing, Visualization. Mary E. Ritchey: Conceptualization, Methodology, Writing – Original Draft, Writing - Review & Editing, Visualization, Funding Acquisition. David D. Dore: Conceptualization, Formal Analysis, Methodology, Investigation, Writing – Original Draft, Writing - Review & Editing, Visualization, Supervision, Funding Acquisition.

## Ethics

Confidentiality and deidentification of patient records were always maintained. All analyses were performed in accordance with applicable laws and regulations.

## Disclosures

**MK** is an employee of Currax Pharmaceuticals, LLC. **CRM, DB, DDD, EM, HW, JS, ML**, **MS**, and **SS** are employees of Exponent, Inc. **CLL, CRM, DB, DDD, EM, HW, JS, KLL, MER, ML**, **MS**, and **SS** were contracted by Currax Pharmaceuticals, LLC, to conduct this study. **SVW** was contracted by Exponent, Inc., and has consulted for Veracity Healthcare Analytics and MITRE Corporation, an FFRDC for the Centers for Medicare and Medicaid Services, for unrelated work. **KLL** is employed by the University of Massachusetts Chan Medical School for unrelated work. This work was governed by a Publications Committee chaired by **DDD** and whose membership included representatives from Currax Pharmaceuticals, LLC. Exponent, Inc., oversaw the study conduct and retained independence in the scientific interpretation of results, reporting, and final wording of any resulting publications.

## Data sharing statement

Data used in this study were supplied by Arcadia Solutions, LLC, as part of one or more research databases. Any interpretation or conclusion based on these data is solely that of the authors and not Arcadia or its third-party licensors. The data use agreement governing this study does not allow authors to share the data, but interested parties can learn more about accessing Arcadia data by visiting https://arcadia.io/.

## Declaration of artificial intelligence

During the preparation of this work, the authors did not use artificial intelligence technologies.

## Source of funding

Funding for this study was provided by Currax Pharmaceuticals, LLC. The study sponsor funded this retrospective database analysis and provided legal and intellectual property approval. Beyond the study sponsor employee authors, the study sponsor did not influence the design and conduct of the study; collection, management, analysis, and interpretation of the data; preparation, review, or approval of the manuscript; or decision to submit the manuscript for publication.
