# Supplementary material for: Cardiovascular safety of fixed-dose extended-release naltrexone/bupropion in clinical practice
Source: Obes Pillars. 2025 Feb 17;13:100169. doi: 10.1016/j.obpill.2025.100169 (PMC11919370; doi:10.1016/j.obpill.2025.100169)
Supplement: Multimedia component 1 [file mmc1.docx]

**Supplementary Material**

**Supplemental Table 1.** Exclusion criteria

| **Exclusion criteria** |
| --- |
| <18 years of age |
| ≥1 recorded ICD-9/10 diagnosis for epilepsy, bulimia, or anorexia |
| Record of weight-loss procedure CPT codes |
| Record of a prescription or dispensing of an opioid medication |
| Prescription/dispensing of NB-ER and lorcaserin on the same day of initiation |

CPT, Current Procedure Terminology; ICD, International Classification of Diseases; NB-ER, fixed-dose extended-release combination of naltrexone and bupropion.

**Supplemental Table 2**. Covariates assessed for each patient

| **Covariates** |
| --- |
| **Baseline demographics** |
| Age |
| Sex |
| Race |
| Ethnicity |
| Smoking status |
| BMI, kg/m^2^ |
| **Comorbidities** |
| AMI |
| Ischemic stroke |
| Hemorrhagic stroke |
| Dyslipidemia |
| Type 2 diabetes mellitus |
| Transient ischemic attack |
| Hypertension |
| Heart failure |
| Unstable angina |
| Peripheral vascular disease |
| Other heart diseases |
| Chronic kidney diseases |
| Liver impairment |
| Chronic obstructive pulmonary disease |
| Obstructive sleep apnea |
| Substance abuse |
| Neuropsychiatric conditions (eg, bipolar disorder, MDD, anorexia nervosa, bulimia) |
| **Concomitant medications contraindicated with NB-ER** |
| Selective serotonin reuptake inhibitors |
| Tricyclic antidepressants |
| Antipsychotics |
| Beta-blockers |
| Type 1C antiarrhythmics |
| Anticoagulants |
| Antiplatelets |
| **Medications that affect CV outcomes** |
| Antihypertensives |
| Statins |
| Antihyperglycemics |
| Cancer therapeutics |
| **Laboratory tests and vital signs** |
| BMI (calculated from weight and height if not available) |
| Blood glucose |
| Hemoglobin A1C |
| High-density lipoprotein |
| Low-density lipoprotein |
| Total body cholesterol |
| Triglycerides |
| Resting heart rate (beats per minute) |
| Systolic blood pressure |
| Diastolic blood pressure |
| Estimated glomerular filtration rate |
| Serum creatinine |

AMI, acute myocardial infarction; BMI, body mass index; CV, cardiovascular; MDD, major depressive disorder; NB-ER, fixed-dose extended-release combination of naltrexone and bupropion.
